# P Selectins and immunological profiles in HCV and *Schistosoma mansoni* induced chronic liver disease

**DOI:** 10.1186/1471-230X-14-132

**Published:** 2014-07-28

**Authors:** Mahmoud M Kamel, Shawky A Fouad, Maha MA Basyoni

**Affiliations:** 1 Department of Clinical Pathology, National Cancer Institute Cairo University, Cairo, Egypt; 2 Department of Internal Medicine, Faculty of Medicine, Cairo University, Cairo, Egypt; 3 Department of Medical Parasitology, Faculty of Medicine, Cairo University, Cairo, Egypt

**Keywords:** HCV, Schistosomiasis *mansoni*, Activated platelets, CD62, Lymphocyte activation

## Abstract

**Background:**

Hepatitis C virus (HCV) and *Schistosoma mansoni* are major causes of chronic liver disease (CLD) in which immune alteration is common. Recent studies suggested that certain platelets and lymphocytes activation markers may have an impact on progression of CLD. This study aimed to evaluate the potential of platelets and lymphocytes activation molecules expression on the pathogenesis of CLD in distinct or concomitant chronic HCV and schistosomiasis *mansoni* infections.

**Methods:**

The study populations were divided into group-I: patients with chronic schistosomiasis *mansoni*, group-II: HCV patients without cirrhosis, group-III: patients with combined liver diseases without cirrhosis, group-IV: patients with chronic HCV and liver cirrhosis and group-V: Age and sex matched healthy individuals as normal controls. All groups were subjected to full clinical evaluation, ELISA anti-HCV antibodies screening, parasitological examination for diagnosing *S. mansoni* and flow cytometry for lymphocyte (CD3, CD4, CD8, CD19, CD22, & CD56) and platelets activation (CD41, CD42 & CD62P (P- selectins)) markers.

**Results:**

The platelet count was significantly decreased in HCV and/or *S. mansoni* patients. The total T-lymphocytes and T-helper cells were significantly reduced, while T-cytotoxics were increased. The patients possessed a significantly higher platelets activation marker; CD62P (P-selectins) and higher mean fluorescent intensity (MFI) positivity. There were considerable correlations between platelets count and both of CD62P and MFI.

**Conclusion:**

Our Findings suggest an increased expression of certain platelets and lymphocytes activation markers in chronic HCV and *S. mansoni* induced CLD that may have a role in disease progression.

## Background

It is becoming esteemed that platelets activity extends beyond ensuring the integrity of the vasculature to comprise defense mechanisms against invading pathogens [1]. Despite their small size and a nucleate status, platelets have diverse roles in vascular biology. In some perspectives, platelet immune functions are protective, whereas in others platelets contribute to adverse inflammatory outcomes [2]. Platelet dysfunctions are commonly associated with CLD and the paradigmal platelet function test has been reported to be abnormal in 2.5–42% of cirrhotic patients [3]. Platelets are activated in liver cirrhosis, hepatocellular carcinoma, schistosomiasis and HCV infections [4]. Thrombocytopenia is frequently found in patients with CLD and cirrhosis. The precise mechanism of thrombocytopenia is unknown; however, several theories have been proposed including decreased thrombopoietin production and accelerated platelet destruction caused by hypersplenism [5].

It is noteworthy that activated platelets have anti-inflammatory properties related to the interaction between CD40L and CD40 and exert a hitherto undescribed immunoregulatory action by enhancing IL-10 production and inhibiting TNF-α production by monocytes [6]. In the complex cytokine network, IFN-ɣ, IL-12, IL-4, and IL-10 play an important role in responses to viral infections by regulating the Th-1/Th-2 balance [7]. The fibrotic mechanism of *S. mansoni* infection tightly acorrelates with high IL-13 and low IFN-γ/IL-10 [8]. Platelet membrane has a large number of glycoproteins that are essential for their normal functioning. Some glycoproteins are present in the resting state as well as after stimulation e.g. CD41, CD42 and CD61. Fibrinogen receptors, CD62p (P-selectin) and CD63 are neoepitopes that appear only on the surface of activated platelets. CD36 induces platelets activation with CD62 expression and their adhesion on leukocytes due to CD62 and CD162 interactions [9]. P-selectins mediate interaction between endothelium, platelets and leucocytes by phosphorylation of histidine residues of the molecule [6]. Of the three known E, L and P-selectins, P-selectins were found to have a critical role in the progression of CLD caused by schistosome parasites. P-selectin is widely thought to promote inflammatory reactions by facilitating leukocyte recruitment. However, it was surprisingly found that mice with targeted deletion of the P-selectin gene (PsKO mice) developed unpolarized type 1/type 2 cytokine reactions and vigorously enhanced liver pathology following infection with the type 2-promoting *S. mansoni*[10]. The ligand for P-selectin, P-selectin glycoprotein ligand-1 (PSGL-1), is expressed on subsets of activated effector T cells and is believed to be essential for the movement of CD4- positive T cells into inflamed tissues [11]. Nonetheless, the extent to which selectins regulate the movement of leucocytes to visceral organs and the contribution of selectins to the regulation of chronic type 2 cytokine dependent liver disease remain relatively unclear. Consequently, this study aimed to assess the potential expression of certain lymphocytes and platelets activation molecules in chronic HCV and/or schistosomiasis *mansoni* infections and their possible roles in progression of CLD.

## Methods

### Ethical approval

This study was conducted in compliance with the Helsinki Declaration and was approved by ethical committee of Faculty of Medicine, Cairo University. (Archiving number; 15/2013).Written informed consents were obtained from all participants.

### Subjects

Eighty seven patients in addition to twenty healthy subjects were selected from the Internal Medicine Department, Kasr AL-Aini Faculty of Medicine, Cairo University during the period from May 2013 to December 2013. The study population was divided into 5 groups. Group-I: 21 patients with hepatic schistosomiasis as evidenced by positive serology and portal tract thickening (grades I-III) by ultrasonography (14 males and 7 females). Group-II: 18 patients with chronic HCV infection without cirrhosis (10 males and 8 females). Group-III: 23 patients with concomitant hepatic schistosomiasis *mansoni* and chronic HCV infections without cirrhosis (17 males and 6 females). Group-IV: 25 patients with chronic HCV and liver cirrhosis (14males and 11females). Group-V: 20 healthy individuals as controls (12 males and 8 females).

### Exclusion criteria

Patients with hepatitis B virus (HBV), malignancy including hepatocellular carcinoma (HCC) or renal, cardiopulmonary or autoimmune disorders and pregnant women were excluded from the study.

### Methods

All participants in the current study were subjected to full history taking (including contact with canal water) and clinical examination in addition to the following investigations:

### Abdominal ultrasound

To assess the hepatic physical condition including the grading of portal tract thickening in schistosomiasis *mansoni* positive patients and the extent of liver cirrhosis.

### Laboratory investigations

1. Complete Blood Count (CBC): Was measured by Sysmix K-21 automatic cell counter (Japan).

2. Liver function tests: Serum levels of aspartate transaminase (AST), alanine transaminase (ALT), albumin, total and direct bilirubin were done using Integra-400 (Roche-Germany). Prothrombin concentration was estimated using Fibrintimer (Roche- Germany).

3. Serological Screening for HBV & HCV: HBV markers and HCV antibodies were assayed by EIA (COBAS-Amplicore, Germany).

4. Qualitative assessment of HCV-RNA by PCR using a commercial kit (Roche Diagnostic, Branchburg, NJ) according to the manufacturer's instructions.

5. Diagnosis of Schistosomiasis *mansoni*: Direct wet mount stool slides were examined in saline and iodine preparations. Concentration slides were prepared using formal-ether concentration technique (FECT) with physiological saline and examined [12]. ELISA sero-immunological detection of anti-*Schistosoma* IgG antibodies was done by indirect ELISA technique [13] using a specific detection kit (Sigma, St. Louis, MO, USA) where microtitration plates were sensitized using *S.mansoni* soluble egg antigen according to the manufacturer's recommendations. In addition, sera of all subjects were screened for *S. mansoni* circulating antigen using sandwich ELISA technique as described previously [14]. All experiments were performed in triplicate and data represent mean values. The cut-off value was calculated as the mean absorbance value of the negative controls plus three standard deviations. A sample was considered positive when the absorbance of the three measurements was greater than 0.114.

6. Flow-Cytometeric Analysis: For immunological parameters, platelets rich plasma (PRP) was separated and freshly tested using fluorescein isothiocyanate (FITC) and phycoerythrin (PE) conjugated monoclonal antibodies (moAbs) (BD Biosciences. Com, Pharmingen TM). EDTA blood was labeled with 10 μl specific moAbs in 3 tubes. The first tube, for T-cell, contained CD3-peridin chlorophyll protein (PerCP), CD4–fluorescein isothiocyanate (FITC) and CD8-phycoerythrin (PE). The second tube, for NK-cells, contained CD16/CD56-PE and CD3-FITC. The third tube, for B-cells, contained CD19-FITC and CD22-PE (all Becton Dickson, San Jose, Calif). A non-specific isotype control was used in each sample. All antibodies were of IgG1k isotype. Flow cytometer Epics® Elite "Coulter" system was used for analysis. Results were expressed as a specific percentage of positive markers, calculated by subtracting the non-specific fluorescence of the isotype control from the specific fluorescence of the moAbs [15]. For estimation of platelets activation, 250 μl EDTA blood was diluted and mixed 1:1 in Hepes-buffer. *Thrombin receptor activating peptide* (TRAP) (Bachem, Germany) was added at a final concentration of 5 μm and samples were incubated for 10 min at room temperature and fixed with one volume 1% formaldehyde. A further inactivated sample was kept for estimation of the baseline fluorescence intensity. At the end of the activation period 30 μl of fixed platelets were washed with Hepes-buffer by centrifugation for 5 min at 750 g. Platelet sediment was resuspended in 200 μl Hepes-buffer and incubated with 10 μl FITC-anti-CD62P (CD62P-FITC, DAKO, USA) and 10 μl PE-anti CD42b antibody (CD42b-PE, DAKO, USA) in darkness at room temperature for 30 min. Sediment labeled platelets were analyzed in a FAC-Scan cytometer (Becton Dickinson, USA). Acquisition and processing of data from 5000 platelets were carried out with CONSORT software. Binding of FITC-labeled antibodies to the surface of stimulated platelets (CD42b positive) is used for expression of CD62P in the form of mean fluorescence intensity (MFI).

### Statistical analysis

The data was coded and entered using the statistical package SPSS version 15. The data was summarized using descriptive statistics: mean and standard deviation. Statistical differences between groups were tested using Chi-Square test for qualitative variables, independent sample t-test with multiple comparisons post-hoc for quantitative normally distributed variables and nonparametric Mann–Whitney test and Kruskal–Wallis test for not normally distributed quantitative variables. Two-way ANOVA test was used to compare between variance of each marker levels among four different infected groups and the controls and P value <0.05 is considered significant.

## Results

**Figure 1:**
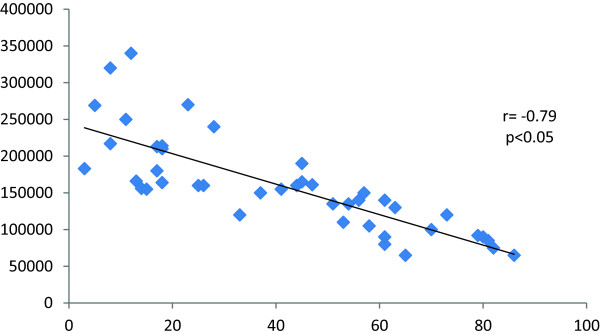
Correlation between platelet count and CD62P%.

**Figure 2:**
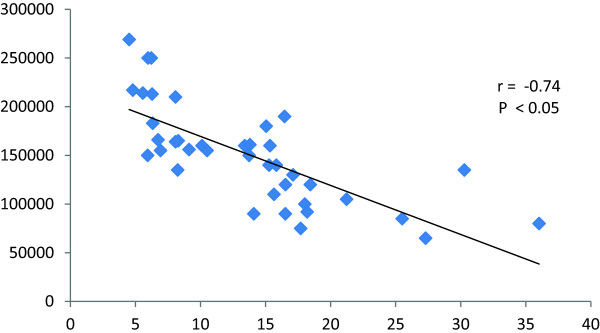
Correlation between platelet count and MFI.

In the present study, subjects were divided into 5 groups. Group-I: Twenty one patients with schistosomiasis *mansoni* (14 males, 7 females) (mean age 48.4 ± 3.9 year). Group-II: Eighteen patients with HCV infection (10 males, 8 females) (mean age 52.4 ± 4 year). Group-III: Twenty three patients with concomitant schistosomiasis *mansoni* and HCV infections (17 males, 6 females) (mean age 56.5 ± 2.7 year). Group-IV: Twenty five patients with chronic HCV and cirrhosis (14males and 11females) (mean age 58 ± 5.76 year) and group-V: Twenty healthy controls (12 males and 8 females) (mean age 46.8 ± 2.5 year). Haematological findings revealed a significant thrombocytopenia in all infected groups in comparison to the control one (P < 0.05) with insignificant (P > 0.05) low Hb concentrations and WBC counts (Table 1). Immunologically, there is a significant reduction in the total T-cells (CD3+%) in all infected groups (P < 0.05) compared to the control group. In addition, a significant reduction in the percentage of the Th-cells (CD4+%) was observed in all infected groups (P < 0.05). On the other hand, the percentage of Tc-cells (CD8+%) was higher in all infected groups (P < 0.05) as shown in Table 2. Regarding B and NK cells, a significant increase in CD19, CD22 (B-cell markers) and CD56 (NK-cell marker) in all infected groups (P < 0.05) compared to the control group (Table 2). The platelet count was significantly decreased (P < 0.05) in all infected groups that was more evident in group-IV (Table 3). The percentages of CD41 and CD42 platelet activation markers were reduced in all infected groups compared to the control group, however, these values were statistically significant in group III & IV (for CD41) and in group II, III & IV (for CD42). The positivity of CD62P (P-selectin) was 12.5 ± 1.9 in the control group while the values were 28.9 ± 4.3, 48 ± 5.2, 67.6 ± 4.4 and 73.4 ± 6.1 in group I, II, III &IV respectively (P < 0.05) with significant increased platelet activation that was mostly significant in group-IV followed by group-III. The MFI in group I, II, III and IV were 12.8 ± 1.4, 15.5 ± 2.5, 17.8 ± 2 & 22.2 ± 1.6 respectively. All were significantly higher (P < 0.05) than the control group (5.9 ± 0.3) (Table 3).The correlation between platelet count and platelet activation revealed an inverse correlation (r = -0.79) (P < 0.05) of the platelet count and the CD62P%. The platelet count was also inversely correlated with the MFI measured by the flowcytomtery (r = - 0.74) (P < 0.05) (Figure 1 and Figure 2).

**Table 1:** Age and laboratory findings of different groups

	**Group I (Schistosomiasis)**	**Group II (HCV)**	**Group III (Schist. & HCV)**	**Group IV (HCV with cirrhosis)**	**Group V (Controls)**
**Age**	48.4±3.932	52.0±4.027	56.5±2.68	58±5.76	46.8±2.458
**Total bilirubin**	0.67±0.22	1.2±0.44	1.1±0.56	2.1±1.7	0.58±0.32
**ALT**	30±2.4	51±6.7	49±7.1	35±4.9	27±5.4
**AST**	29±6.5	37±9.6	38±4.5	39±5.2	25±7.8
**PC**	89.3±2.1	87.2±3.6	85±4.2	51.3±7.3	96.2±1.6
**Albumin**	4.2±1.1	3.8±1.7	3.9±1.3	2.8±1.2	4.8±1.1
**Hb**	11.3±0.5	11.5±0.5	11.2±0.5	10.5±0.7	12.8±1.1
**WBC**	7381±694	6090±521	5550±567	3250±1650	7500±744
**Platelet count**	161±9,3	135±1,5	134±9,6	112±2,5	275±20
**Neutrophils%**	48.4±2.9	50.9±2.3	51.7±2.3	52.1±4.1	53.1±2.7
**Lymphocytes%**	38.2±3.1	34.3±3.1	36.3±2.1	37.5±2.6	33.3±2.6
**Monocytes%**	8.7±0.8	10.5±1.4	7.9±0.4	10.0±1.9	8.2±0.7

Values are expressed as mean ± SD.

**Table 2:** Immunological profiles of different groups

	**CD %**				
	**Group I**	**Group II**	**Group III**	**Group IV**	**Group V**
**CD3**	48.2±1.9^b^	53.7±1.7^b^	48.7±1.3^b^	44.7±3.1^b^	63.8±1.3^a^
**CD4**	25.7±1.2^b^	27.0±1.5^b^	25.5±1.4^b^	24.5±1.8^b^	42.9±0.9^a^
**CD8**	26.3±1.3^a^	25.8±1.6^a^	25.2±0.8^a^	24.5±1.4^a^	20.2±0.7^b^
**CD19**	17.2±0.9^a^	18.4±1.3^a^	17.7±0.7^a^	18.1±1.1^a^	14.3±1.0^b^
**CD22**	16.5±0.9^a^	17.9±1.1^a^	17.4±0.6^a^	18.7±0.9^a^	13.8±0.8^b^
**CD56**	12.8±0.7^a^	13.6±1^a^	14.9±0.5^a^	15.2±0.7^a^	9.7±0.6^b^

Values are expressed as mean ± SE.

Statistically significant values (P<0.05).

Means followed by the same superscript letter within the same row means non-significant variation (P>0.05) in relation to each other, but statistically significant in relation to the control group.

**Table 3:** Platelet counts, markers and activation in different groups

	**Group I**	**Group II**	**Group III**	**Group IV**	**Group V**
**Platelet count**	161±9,3^b^	135±1,5^c^	134±9,6^c^	112±2,5^d^	275±20^a^
**CD62%**	28.9±4.3^d^	48.0±5.2^c^	67.6±4.4^b^	73.4±6.1^a^	12.5±1.9^e^
**MFI**	12.8±1.4^c^	15.5±2.5^b^	17.76±2.0^b^	22.2±1.6^a^	5.9±0.25^d^
**CD41%**	91.9±0.6^ab^	91.9±0.8^ab^	90.4±1.1^b^	87.4±4.0^b^	94.1±0.7^a^
**CD42%**	92.2±0.7^ab^	91.5±0.7^b^	91.1±0.9^b^	90.2±1.5^b^	94.7±0.6^a^

Values are expressed as mean ± SE.

Statistically significant values (P<0.05).

Means followed by the same superscript letter (a,b,c,d or e) within the same row means non-significant variation (P>0.05) in relation to each other, but statistically significant in relation to the other groups and to the control group.

Mean followed by (ab) superscript means that this group is statistically insignificant to either groups with superscript (a) and superscript (b).

## Discussion

This study aimed to characterize the expression of platelets and lymphocytes activation molecules in CLD in distinct or simultaneous chronic HCV and schistosomiasis *mansoni* infections. Patients with CLD are suffering from impairment of immune function due to significant reduction of both CD3+ and CD4+ lymphocytes. This reduction was found to be correlated with severity of liver disease [16].

In agreement with that, the current study revealed a significant decrease in CD3+ and CD4+ cells in HCV, *S. mansoni* infected groups, concurrent dually infected individuals and those with liver cirrhosis. These findings agreed with the fact that, the absence of an adequate CD4 + cell response is associated with incomplete HCV eradication by memory CD8+ cells and failure to resolve HCV infection [17]. Additionally, low CD4 + cells counts are also associated with increased rates of liver fibrosis [18]. Recently, data show that HCV-core protein induces a suppressor phenotype in CD4+ T-cells. HCV-core expressing CD4+ T-cells showed an anergic phenotype, being unresponsive to T-cell receptor (TCR) stimulation and being able to suppress polyclonal CD4+ and CD8+ T-cell activation [19].

In a bit similar mechanism, *S. mansoni* appeared to utilize the activities of CD4+ T-cells to assist the parasite development and fecundity [20]. This was explained by Kullberg and his colleagues who mentioned that *S. mansoni* implied a Th2-cytokine-mediated immunopathogenesis with impairment of the Th1-dependent immune response involving both CD4 + T-cell delayed-type hypersensitivity responses and CD8+ T-cell antiviral effector functions [21].

In the present study, we reported an increase in the percentage of Tc-cells (CD8+) in all infected groups. This was confirmed by Manfras et al. who stated that the increased oligoclonality of CD8+ lymphocytes is associated with increased fibrosis and reduced responses to antiviral therapy [22]. On the same line, Li et al. found that the ratio of CD4+/CD8+ was significantly decreased in *Schisotosoma-*infected patients and those with parenchymal fibrosis [23].

Also, our study revealed a significant increase in the B-cell markers (CD19& CD22) observed in patients with HCV infection. These results are consistent with previous studies which explained that HCV can replicate in CD19+ B-cells [24] as HCV envelope protein-E2 binds the CD81 molecule that is expressed on hepatocytes and various cell types including B-cells [25]. Moreover, recent evidence reported that at least one HCV replication marker was found in 50% and 30.8% of CD3+ and CD19+ cells respectively. The authors added that the highest percentage of cells harboring the viral markers in a single specimen was observed in CD3+ (2.4%), then in CD19+ (1.2%) cells [26]. Previous studies suggested the hypothesis of persistent stimulation of B-cells by viral antigens that could be responsible for polyclonal and later to monoclonal expansion of B-cells [27,28]. Nevertheless, B-cells cannot support HCV replication in certain HCV strains but can bind HCV and trans-infect hepatocytes [29].

In schistosomiasis, it was reported that the mean percentage of circulating CD19+ B-cells was significantly high in *S. mansoni*–infected patients [30]. This could be explained through studies carried on schistosomiasis *mansoni*-infected B cell-deficient mice, which revealed more extensive hepatic granulomas that were explained by the role of B-cells in the down modulation of liver pathology through promoting Th2-type responses [31,32]. In addition to CD19, we reported that CD22 was highly expressed in HCV& cirrhotic patients. CD22 is known as an inhibitory receptor specifically expressed on B-lymphocytes. Eosinophils are known to express the receptor for IL-4, which induce CD22 on B-cells. CD22 is functionally involved in regulating GI eosinophil levels [33]. To our knowledge, the current study is one of the earliest reports demonstrating high expression of the pan B-cell marker-CD22 in *S.mansoni* infected patients.

In the present study, we revealed that patients with chronic HCV showed an increase in CD56+ NK-cells in their peripheral blood. What is more is that, the percentage of NK-cells (CD56%) showed a significant increase in all infected groups. These results are adding to the several arguments about the alterations of the peripheral NK-cells for patients chronically infected with HCV. First, previous studies have shown that chronic HCV infection is allied with diminished NK-cell frequency and function in the peripheral blood and in the liver [34]. Moreover, Golden-Mason et al. reported that the reduction in CD56+ NK levels occurred early in acute HCV infection and did not fluctuate over time [35]. On the other hand, two earlier reports did not detect any decrement in peripheral blood NK-cell percentages in HCV infection [36,37]. Furthermore, another study revealed no observed differences between chronic HCV patients and healthy individuals in the number and frequencies of CD56 NK-cells [38]. We proposed a potential elucidation for these variations which could be selective trapping of CD56 NK-cells in the liver in case of HCV infection resulting in alterations of the tissue localization of these cells. Despite this suggestion, there is no strong evidence to support this hypothesis in chronic HCV infection as the ratios of CD56- subsets in blood and liver of HCV patients were very similar [39].

Similarly in schistosomiasis infection, the large numbers of NK-cells induces inhibition of *Schistosoma* development [40].

Patients with advanced liver diseases have a complex hemostatic disturbance and thrombocytopenia [41]. Thrombocytopenia and platelet function abnormalities are commonly found in patients with CLD [42]. In agreement with that the current study revealed significant thrombocytopenia in all infected groups.

Platelets facilitate T-cell adhesion and may influence other functional aspects of T-cells by releasing platelet-factor-4 (PF4) that regulates multiple T-cell activities [43]. It was shown that platelet depletion diminished accumulation of virus specific Tc-lymphocytes and minimized organ damage. On the other hand, platelets seem to enhance the generation of IFN-γ- producing TC-cells [44]. Accordingly, the effect of thrombocytopenia on CLD progression among HCV and/or *Schistosom-* infected patients might be exerted through alterations in T-cell functions and activity.

Additionally, platelets have a role in protection against schistosomiasis through expression of IgE-receptors that are considered as an important defense mechanism in parasitic infection [45]. IgE binding induces platelet release of cytotoxic mediators that subsequently kill *Schistosoma*[41]. Even though IgE is known to be protective against adult stages of *S mansoni*; studies during the chronic stage of infection reported that IgE anti-parasite antibodies have been implicated as protective against the soluble egg antigens (SEA), as it was reported that SEA-IgE antibody level was associated with resistance to reinfection with *S. japonicum*[46].

Similarly, IgE-isotypes to SEA, in 58 patients from Brazil were analyzed, in order to evaluate the patterns of antibodies responses before and after treatment. Before treatment, IgE and IgG4-anti-SEA antibody levels were more elevated. These antibody levels tended to increase after treatment suggesting stimulation of the antibody response owing to the drug effects or antigens exposure due to parasitic stage damage [47].

Chronic HCV infection induces alterations of markers of inflammation and endothelial dysfunction [48]. In addition, the increased level of P-selectin has been proposed as a marker of *in-vivo* platelet activation [49]. Although, a significant positive relationship was reported between an increased serum P-selectin during anti-HCV therapy [48], the current study detected an increase in the positivity of the CD62P (P-selectin) demonstrating an increased platelet activation that was significantly observed in group-IV followed by group-III, group-II then group-I. Such increase in P-selectin in the cirrhotic group compared to the non-cirrhotic and control groups may propose the role of P-selectin in progression of CLD.

The MFI in all infected groups was significantly higher (P < 0.05) than that of the control group (5.9 ± 0.3). An inverse correlations between the platelet count and MFI (r = -0.74) were observed. MFI rate is a numerical data reflecting the severity of antigen expression [42]. These findings were in agreement with a study reported that plasma soluble P-selectin levels were markedly elevated in chronic HCV which correlated directly with serum HCV-RNA and was significantly higher in patients with low platelet counts [50]. Moreover, Panasiuk et al., found elevated P-selectin expression in chronic hepatitis and cirrhosis and they suggested that HCV infection might be directly responsible for the *in-vivo* platelet activation in patients with chronic HCV [16]. On contrary, Wynn et al. concluded that P-selectin exhibits critical anti-inflammatory and anti-fibrotic activity and dramatically inhibit the pathologic tissue remodeling resulted from chronic type-2 cytokine-mediated inflammation [10]. This was ascertained by Laschke et al. who reported that platelet depletion or blockage of the P-selectin receptor was reported to decrease aggregate formation, platelet adhesion and leukocyte accumulation resulting in improved liver functions [43].

During experimental schistosomiasis, the presence of lacto-N-fucopentaose-III (LNFP-III) was demonstrated on SEAs [51]. LNFP-III induces proliferation of splenic B**-**lymphocytes of *S.mansoni-*infected mice to produce IL-10 and thus down-modulate Th1. Interaction between LNFP-III and B-lymphocytes is mediated by P-selectin [52]. In addition, it was found that P-selectin, acts as a decoy-receptor up-regulating IL-13Rα2 in *S. mansoni* infection [10] with subsequent exacerbation of *S. mansoni* associated liver fibrosis due to increased IL-13activity [53]. Also, Liu and his colleagues reported that markedly elevated protein expression of IL-13 was detected in patients with HCV-associated cirrhosis which could elucidate the elevated P-selectins in patients with HCV [54].

In the current study, all groups exhibited decreased expression of CD41 and CD42 which are known to be expressed on the surface of resting and activated platelets. These results could be explained by the hypothesis that platelets microvesicles (PMVs) may transfer specific platelets antigen CD41/CD61 into other cells reducing their expression on activated platelets [55]. Moreover, in case of diseases with immunological disturbances, platelets may serve as targets for anti-platelet antibodies targeting the micromolecules expressed on their surfaces [55,56]. To a certain extent, similar results were reported in a flow cytometric assay for immunophenotyping of PMVs in platelet rich plasma concentration in two groups of patients with and without inflammation. In both studied groups after thrombin stimulation the number of PMVs was decreased, however, the platelets delivered more PMVs expressing activation marker CD62P. The number of PMVs expressing CD41+ CD62P + was only increased in patients without signs of inflammation [56].

## Conclusion

The present study highlights the considerable role of P-selectin in the progression of CLD caused by HCV and *S.mansoni*. By upregulating platelet activation, P-selectin modulates consequences of chronic type 2 cytokine–mediated inflammation. Although selectins frequently are referred to as provocative mediators, it remains indistinct whether this impact results from the direct effect of P-selectin on leukocyte recruitment or it is basically an outcome of the altered host immune reaction. The extent of platelet and lymphocyte activation markers described herein releases a new gap for prognostics predicting the progression of HCV and schistosomiasis *mansoni* induced CLD and consequently, the development of new preventive measures.

## Abbreviations

HCV: Hepatitis C virus; CLD: Chronic liver disease; ELISA: Enzyme linked immunosorbent assay; CD: Cluster of differentiation; MFI: Mean fluorescent intensity; HBV: Hepatitis B virus; HCC: Hepatocellular carcinoma; CBC: Complete blood count; AST: Aspartate transaminase; ALT: Alanine transaminase; EIA: Enzyme immunoassay; PCR: Polymerase chain reaction; RNA: Ribonucleic acid; FECT: Formol ether concentration technique; IgG: Immunoglobulin g; PRP: Platelet rich plasma; FICT: Flurescine isothiocyanate; PC: Phycoerythrin; moAbs: Monoclonal antibodies; TRAP: Hepes-buffer T*hrombin receptor activating peptide*; Hb: Haemoglobin; WBC: White blood cell; TCR: Tcell receptor; PF4: Platelet factor 4; SEA: Soluble egg antigen; LNFP-III: Lacto-N-fucopentaose-III; PMVs: Platelets microvesicles.

## Competing interests

The authors declare that they have no competing interests.

## Authors’ contributions

SF, MK and MB conceived and designed the experiments. MK and MB executed the laboratory work. SF and MB led the writing of the manuscript and analyzed the data. All authors read and approved the final manuscript.

## Pre-publication history

The pre-publication history for this paper can be accessed here:

http://www.biomedcentral.com/1471-230X/14/132/prepub
